# Integrated Sustainable Management of Petrochemical Industrial Air Pollution

**DOI:** 10.3390/ijerph20032280

**Published:** 2023-01-27

**Authors:** Jutarat Keawboonchu, Sarawut Thepanondh, Vanitchaya Kultan, Nattaporn Pinthong, Wissawa Malakan, Mark Gregory Robson

**Affiliations:** 1 Department of Sanitary Engineering, Faculty of Public Health, Mahidol University, Bangkok 10400, Thailand; 2 Center of Excellence on Environmental Health and Toxicology (EHT), OPS, MHESI, Bangkok 10400, Thailand; 3 Department of Plant Biology, School of Environmental and Biological Science, The State University of New Jersey, New Brunswick, NJ 08901-8525, USA

**Keywords:** air pollution, air dispersion modeling, BTX, emission inventory, petrochemical industry, scenario analysis

## Abstract

The emission inventory, emission factor, and spatial concentration distribution of volatile organic compounds (VOCs) from a petrochemical industry (aromatics plant) were intensively evaluated in this study to elucidate the potential sources of BTX emission and their contribution to ambient concentrations. Five emission groups were quantified through direct measurement and emission models. These data were then used as input for the AERMOD dispersion model for the source apportionment analysis. The source to ambient contribution analysis revealed that a wastewater treatment facility and organic liquid storage tank were major contributors accounting for about 20.6–88.4% and 10.3–75.4% to BTX environmental concentrations, respectively. The highest annual ambient concentrations of benzene (B), toluene (T), and xylenes (X) were predicted as 9.0, 2.8, and 57.9 µg/m^3^ at the fence line of the plant boundary, respectively. These findings assist policymakers in prioritizing the appropriate control measures to the right source by considering not just the amount released but also their contribution to ambient concentrations. This study suggested that the wastewater treatment unit should be changed to the closed system which will benefit reduction in its emission (45.05%) as well as effectively minimizing ambient VOC concentration by 49.96% compared to its normal operation.

## 1. Introduction

With rapid growth of urbanization and industrialization, air quality has become worse and more severe to human and environmental health [1,2,3]. Conventional air pollutants include particulate matter (PM), oxides of nitrogen (NO_x_), sulfur dioxide (SO_2_), carbon monoxide (CO), and volatile organic compounds (VOCs), which are known to be released from anthropogenic sources [4,5,6]. As for VOCs, besides their direct impact on health, they are also precursors of secondary air pollutants such as secondary organic aerosol and ground level ozone [7,8,9].

An emission inventory is a crucial tool for estimating the amount of pollutant discharged to the atmosphere. Establishing a VOC emission inventory is also an important route to identifying the sources and characteristics of pollutants. For several years, many studies aimed to develop emission inventories at different spatial scales and in different sectors [10,11,12]. Moreover, the tools can be used to further study the impact of pollutants in terms of concentration and aid the creation of appropriate policies [13,14].

The Map Ta Phut industrial complex, the largest industrial petroleum refinery hub of the petrochemical industry in Thailand, was chosen as a study area. This area is one of the country’s most environmentally toxic hotspots of air pollution [15,16,17]. VOCs are emitted from transportation, industrial production, and operation. For the past several years, little research in Thailand has been carried out to quantify and characterize VOC emission in the area [18]. From local research in the area, an emission inventory was carried out to identify the potential sources for whole industrial complexes and some research monitored the ambient air around the factory and focused on the current situation of the environmental impact on ambient air [19]. However, no policymakers or researchers have evaluated VOC emission associated with different processes within the petrochemical plants. As a result, the development of a systematic emission inventory for the petrochemical industry in the local area has been interrupted. Besides the emission inventory, concentration in the environment is also crucial. Policymakers or researchers may wish to calculate a concentration distribution that is released from sources by using reliable modeling systems in order to make proper suggestions [20,21].

To fill the knowledge gaps, the aromatic plant was selected as a showcase for comprehensive analysis due to its large consumption of both carcinogenic and non-carcinogenic VOCs. Source contribution and spatial distribution of VOCs were also determined to identify the key sources that affect the concentration in the environment. Further, the improvement of VOC control technologies and appropriate mitigation measures were proposed in this study to estimate the emission and concentration reduction.

## 2. Literature Review

### 2.1. Pollutants from Petrochemical Industry

The petrochemical industry is considered a major source of hazardous and toxic air pollutants. It is also a potential source of criteria air pollutants such as particulate matter (PM), nitrogen oxide (NO_x_), carbon monoxide (CO), hydrogen sulfide (H_2_S), sulfur dioxide (SO_2_), and greenhouse gas (GHG) [22,23,24]. Air emissions are released from several processes, including stack and fugitive sources. Besides the direct impact from primary air pollutants emitted from the petrochemical industry, the photochemical reaction of volatile hydrocarbon and oxides of nitrogen contributing to ozone formation is one of the most important air pollution problems worldwide [25].

Hydrocarbons, both aliphatic and aromatic, are the major VOCs emitted from the petrochemical industry. Aromatic VOCs mainly comprise benzene, toluene, and xylenes (BTX). Benzene is also of particular concern since it is a carcinogenic substance [26]. BTX concentrations in some of the world’s industrial, urban, and rural regions are presented in Table 1. The highest concentrations of BTX were observed in the industrial areas, followed by the urban and rural areas, respectively.

Map Ta Phut industrial complex, the largest industrial complex in Thailand and the world’s eighth-largest petrochemical industrial hub and oil refinery, has contributed considerably to the GDP of the country [33,34]. An emission inventory of SO_2_, NO_2_, and some VOCs was developed for the industrial source in this industrial complex. These data are used by policymakers to decide the appropriate air quality policy to be specifically implemented in this industrial area [21,35].

### 2.2. Evaluation of Pollutant Dispersion

To investigate the influence of air pollutants emitted from the source to the atmosphere, air quality and trajectory models are among the most favorable methods. At present, there are several air quality models being used to serve different purposes. AERMOD and CALPUFF are examples of local scale models in which only physical characteristics are explained, while CAMX, CMAX, and WRF-Chem serve the regional scale with chemical reactions added in the calculation. A summary of advantage and limitation of various models is shown in Supplementary Materials, Table S1. The selection of the dispersion model is one of the crucial factors in the accuracy of the output results. Factors that should be considered when selecting the appropriate dispersion model are the characteristics of the boundary domain, type of pollutants, source types, location characteristics, and meteorological characteristics [36]. The AERMOD model has been widely used for many applications such as cement factories, power plants, roadside areas, and industrial complex facilities within local areas [37,38,39], while the CALPUFF model can be utilized to simulate the air dispersion at medium to large scales (up to more than 80 km) [40]. Some studies used the CALPUFF model to predict the concentration and odor dispersion in complex terrain [41,42].

## 3. Materials and Method

### 3.1. Study Area

**Figure 1:**
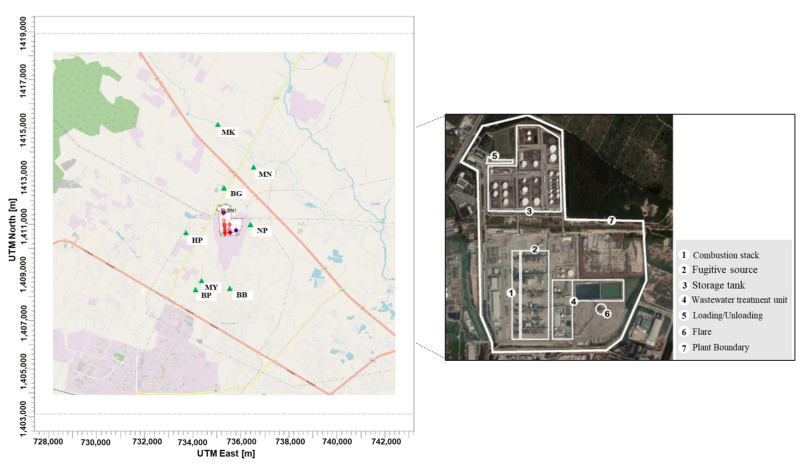
Locations of VOC emission sources.

This study was conducted at the aromatics manufacturing industry located in Map Ta Phut industrial complex. It is a leading manufacturer of aromatic hydrocarbons such as benzene, toluene, and xylenes (BTX), with its business including manufacturing and upstream, intermediate, and downstream distribution of 1.4 million tons of aromatic products per year. The spatial distribution of processes of five major emission sources of VOCs, namely a fugitive source, storage tank, combustion stack, wastewater treatment unit, and loading/unloading facility is illustrated in Figure 1.

### 3.2. Development of VOC Emission Inventory

VOC emissions from the petrochemical industry (aromatic manufacturing) can potentially be released from several production and operation processes, including from equipment dynamic and static sealing point leakage, loss of organic liquid storage and reconciliation, loading and transferring, as well as stack and wastewater treatment units [43]. In the aromatic production processes such as aromatics fractionation units, the raw chemical materials are converted to benzene, toluene, and xylene substances [44], which are the major chemicals used and produced. Therefore, these chemicals were used to represent VOCs in this study. 

Generally, VOC sources from the petrochemical industry can be divided into 5 categories in normal continuous operation as follows: (a) combustion stack, (b) fugitive sources, (c) storage tanks, (d) wastewater treatment units (WWTs), and (e) loading/unloading [22]. All listed sources were covered by this study. The BTX emission data and required characteristics of their sources were obtained from the target factory. The VOC emission inventory was developed by combining top-down and bottom-up approaches. Emission estimating techniques are summarized in Table 2. 

To minimize the uncertainty of the emission factor methods, this study was conducted across direct measurement and emission models to intensively quantify the emission inventory in 2019. Moreover, the emission estimation for business as usual (BAU) were estimated in the worst-case scenario for conservative purposes.

#### 3.2.1. Estimation of Combustion Stack Emissions

For the aromatic processes, the combustion stack was installed in the feed fractionation, isomerization unit and aromatic fractionation unit. The fuel gas and fuel oil were synergistically used for the production processes. Emission estimations of VOC methods for the petroleum refinery were applied to calculate the industrial emissions in the petrochemical industry [45]. The VOC emission rate is normally calculated by using the following Equation (1):

(1) $$E_{j}=\sum_{j}{(Q}_{j}{\;\times \;C}_{j}{\;\times \;h}_{j})$$

where j is the VOC emission source, E_j_ is the annual VOC emission of the source j, Q_j_ is the flow rate of VOC emission from the source j, C_j_ is the VOC emission concentration of the source j [46], and h_j_ is the annual emission hours (8760 times) of source j. 

#### 3.2.2. Estimation of Fugitive Equipment Emissions

Fugitive sources, or equipment sealing point leakage, are representative of the small-scale emission sources throughout the petrochemical industry area. In this study, the VOC emissions from fugitive sources in production processes were obtained from the industrial plant. In normal operation, the released concentration from fugitive leakage was directly measured once per year using a Krypton 10.6 eV photo ionization detector (PID) lamp. The leakage equipment of the aromatic plant comprised liquid valves, liquid pumps, liquid pressure relief devices, connectors or flanges, open-ended lines, sampling connections, and agitation or mixers. The industrial plant set the internal standard concentration for each equipment. When the screening values were detected to be higher than the internal standard, maintenance and remeasurement were performed before recording and reporting the data. The VOC emissions for the process equipment were calculated by using the US EPA correlation equation method [47]. The relative equation was adopted for estimating the emission rate $\left(e_{TOC}\right)$ according to Equation (2):

(2) $$e_{\mathrm{TOC}}=\overset{n}{\underset{i=1}{\sum }}\{\begin{array}{c} e_{0,\;i}\;\left(0\leq \mathrm{SV}<1\right)\\ a\cdot {\mathrm{SV}}_{i}^{b}\;\left(\mathrm{SV}\geq 50,000\right)\\ e_{f,\;i}\;(1\leq \mathrm{SV}<50,000) \end{array}$$

where SV is the screening value at leakage point i [48], $e_{0,\;i}$ is the default zero emission rate at leakage point i (kg/h), a and b are the two variables in the correlation equation (kg/h), and $e_{f,\;i}$ is the pegged emission rate at leakage point i (kg/h). The parametrization scheme of each type of fugitive components is shown in Supplementary Materials, Table S2.

#### 3.2.3. Estimation of Storage Tank Emissions

Storage tanks are important sources of VOCs in the petrochemical industry. VOC emissions can occur from many mechanisms and processes, namely standing losses and loading losses. The US EPA TANKS model is popular for the calculation of VOC emission from storage tanks [49,50,51]. In this study, the method for estimating the emission from storage tanks was performed by using TANKS model version 4.0.9d [52]. The specific parameters consisted of monthly meteorological data, physical characteristics of tanks, monthly throughput data, actual chemical composition, and treatment and removal systems. The aromatic plant has many vertical fixed roof tanks and internal floating roof tanks in normal operation. Their emission rates were estimated under the assumption that the internal shell condition is gunite lining. However, VOCs from the storage tank are not directly released into the atmosphere but are connected to the vapor recovery unit (VRU) system with 95% collection and control efficiency.

#### 3.2.4. Estimation of Wastewater Treatment Unit (WWT) Emissions

Wastewater and effluent water from industrial processes entering the wastewater treatment unit (WWT) of the petrochemical industry mainly come from raw material tanks, production process, and the tank farm. Generally, WATER9 is used to estimate air emissions from site-specific water treatment plants (including the prediction of biodegradation and sludge sorption of organics) for common wastewater treatment units, including drains, close/open sumps, weirs, pump stations, clarifiers, and activated sludge units [53,54]. To estimate VOC emissions from wastewater treatment unit in this study, the WATER9 model was utilized [55]. The specific data of wastewater and unit characteristics such as solid and oil contents, effluent temperature, and volumetric flow rate were inputted to estimate the BTX emission rate from wastewater treatment units.

#### 3.2.5. Estimation from Loading/Unloading Emissions

Transportation and storage in the petrochemical industry pertains to the movement of petrochemical products to other processes and storage areas. Emissions from loading/unloading petrochemical liquid with the reduction efficiency can be estimated using the following Equation (3):

(3) $$L_{L}=12.46\frac{\mathrm{SPM}}{T}\left(1-\frac{\mathrm{eff}}{100}\right)$$

where L_L_ is the loading loss (kg/m^3^) of loaded liquid [56], S is a saturation factor (Supplementary Materials, Table S3), P is the true vapor pressure of loaded liquid (kPa), M is the molecular weight of vapors (g/mol), and T is the temperature of bulk liquid load (K).

### 3.3. Emission Factors

An emission factor, the quantity of VOCs released to the air with an associated activity is a key parameter in the calculation of atmospheric pollutant emission, and it directly influences the accuracy of the emission inventory. Emission factors are defined as the amount of pollutant emitted per ton of product yield [57]. According to the VOC emission inventory, the emission factor of the target industry can be used to establish reliable VOC emissions from the petrochemical industry. The VOC emission factors (EFs) for each source were calculated according to Equation (4):

(4) $${\mathrm{EF}}_{i}=\frac{E_{i}}{A_{i}}\;,$$

where EF_i_ is the emission factor of source i, kg VOCs/kg products. E_i_ is the emission load of source i (kg/y), and A_i_ is the activity level (the amount of product yield), (kg/y).

The total emission factor for the petrochemical industry can be achieved by summing the emissions from each source and combining them using the following Equation (5):

(5) $$\mathrm{EF}=\frac{\sum_{i=1}^{n}E_{i}}{A},$$

where EF is the total emission factor for the petrochemical industry (g VOCs/kg products). A is the amount of product yield (t/y), and n is the number of emission sources.

### 3.4. Mathematical Dispersion Modeling

Emission of VOCs from each individual source was analyzed together with local meteorological data using the AERMOD dispersion model (Lakes Environmental, version 9.8.3) to predict the ground level concentration of VOCs. AERMOD was chosen in this study since it is widely used for industrial application, and it is designated as a regulatory model by the US EPA [58]. The AERMOD model is one of the most popular tools for air quality impact assessment at a local scale (within 50 km of the source). The AERMOD model was widely used to estimate the dispersion of air pollutants both in terms of the pollution concentration and the odor nuisance in many studies. The AERMOD model has been utilized to forecast the air quality in the environment and is also used at interested receptor sites as a sensitive location to further simulate the control and management policy. Ma et al. (2013) predicted the average value of SO_2_ and NO_x_ in near-future simulations. They found that the policymaker must announce the air quality control measure before the ambient concentrations exceed the air quality standards in the coming year [59]. Abdel-Gawad et al. (2022) measured and evaluated long- and short-term health impacts to predict the ground level concentration of SO_2_, mercury, arsenic, and chromium VI by using the AERMOD model within a 30 km radius of the studied cement industry. The finding revealed that meteorological data and topographical data were the important factors in predicting pollutant dispersion [60]. In Thailand, Seangkiatiyuth et al. (2011) applied the model to study the environmental impact assessment of NO_2_ from the cement complex. The simulation results can help policymakers to identify the areas of maximum concentration [61]. For example, Kultan et al. (2022) utilized the AERMOD model to predict the odor of industrial VOCs from automobile manufacturing. The key contributor to the smell and appropriate measures in managing industrial air pollution were identified [62]. 

In this study, the centroid of the studied domain was configured at UTM 735410 m N and 1411025 m E. The dimensions of the map were 14 × 14 km. The AERMOD model consists of three main data inputs: the mass emissions data that are obtained from the factory; the terrain heights from the high-resolution NASA Shuttle Radar Topography Mission (SRTM) database, which is the terrain preprocessor of AERMOD; and the 1-year (1 January 2019 to 31 December 2019) meteorological data generated by AERMET, which is the meteorological preprocessor of AERMOD. The hourly surface and upper air data were arranged in the format of an SFC file and a PFL file. The upper air data consisted of atmospheric pressure heights, temperature, dew point temperature, wind speed, and wind direction, while the hourly surface data also consisted of wind speed, wind direction, ambient temperature, cloud cover, and ceiling height. These data were acquired from direct measurement of the 100 m meteorological mast operated by the Thai Meteorological Department.

**Figure 2:**
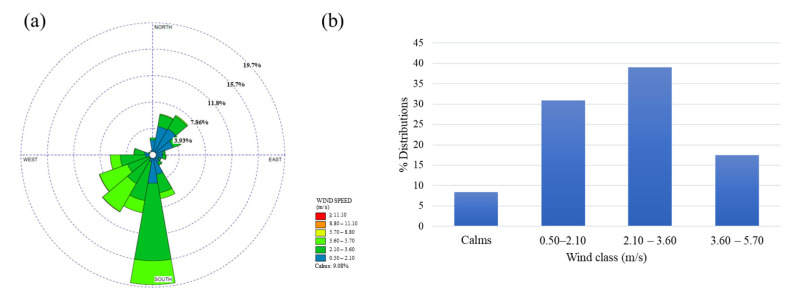
(**a**) Wind rose plot of study area; (**b**) % distribution of wind classes.

The major wind direction that prevailed from the south and the west direction is shown in Figure 2a. The velocity of the wind was generally moderate, with about 9.1% of calm wind (wind speed < 0.5 m/s) occurrence for the whole year measurement. The local winds were accumulated in three categories. They are sorted as follows in Figure 2b: 2.10–3.60, 0.50–2.10, and 3.60–5.70, with a contribution of 40.4, 32.6, and 17.8%, respectively.

### 3.5. Scenario Analysis

Scenario analysis was conducted in this study to assess the success of current and proposed control technology at the factory to control VOC emission with the goal to reduce the ambient concentration. These scenarios, including past work, the existing scenario, and the plans for controlling of VOC emission, are listed in Table 3. The emission and concentration in each scenario (Supplementary Materials, Tables S4–S6) were evaluated according to the identical methods of business as usual (BAU). Scenario analysis of VOC concentration was conducted by using the average concentration of different receptor sites to represent the overall concentration in the study domain.

Scenarios No. 1 and 2 were set to predict the emission and concentration without any policy intervention and when the activated carbon has been installed, respectively. The current situation was represented by scenario No. 3 of BAU when the vapor recovery unit was installed to treat VOC emission from storage tanks. Three mitigation measures were suggested: in scenario No. 4, by assuming the direct measurement on the monitoring station at VRU; in scenario No. 5, by improving the tank characteristics of the internal floating roof tank (IFRT) from gunite lining to light rust; and in scenario No. 6, by enclosing the open sump in the wastewater treatment unit.

## 4. Results and Discussion

### 4.1. Emission Inventory in the BAU Scenario

**Figure 3:**
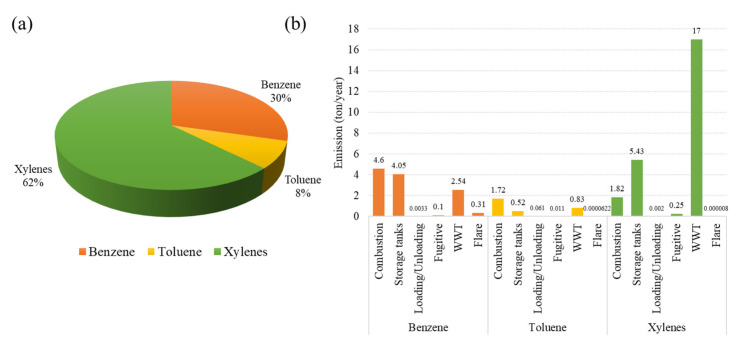
(**a**) VOCs (BTX) emission contribution from the petrochemical industry; (**b**) source contribution to the VOCs (BTX) from the petrochemical industry.

In the study, a VOC emission inventory of benzene, toluene, and xylenes (BTX) from the target industry was intensively quantified through the methods described in Section 2. Figure 3a,b illustrate the emission characteristics and contribution of BTX emitted from different sources of the petrochemical industry. Overall, the largest proportion of VOC emissions was shared by xylenes with the total amount of 24.50 tons/year, accounting for 62% of the total VOC emission. The wastewater treatment unit was found to be the major contributor, accounting for 69% of xylene emissions, followed by storage tanks (22% of xylene emissions). Benzene emission was about 11.62 tons in the base year, where the combustion source was the largest contributor, contributing 41% of the total benzene emissions. The storage tanks shared 36% of the benzene emissions, followed by wastewater treatment units; fugitive sources accounted for 22% and 1% of the total benzene emissions. As for toluene, the total emissions were 3.14 tons/year, of which the production of VOCs, the combustion sources, wastewater treatment units, and storage tanks accounted for 55%, 26%, and 17% of its total emissions, respectively. These measurements were in agreement with the other previous study, regarding the whole picture of the Map Ta Phut industrial complex area. Xylene emissions from industrial processes were found to comprise the largest proportion (more than 30%) among BTX [19]. Moreover, according to the inventory, the BTX emissions are consistent with the industrial production capacity. Regarding the growing trend (7.2% growth rate in 2022) in the demand of petrochemical intermediates, paraxylenes are the main product yield from aromatic manufacturing [63].

Results from this study revealed that wastewater treatment units and storage tanks are the dominant VOC emission sources. This was mainly associated with the chemical throughput that passed through these sources in production processes. VOC emission from wastewater treatment units was quantified by using the US EPA’s WATER9 model, which is a comprehensive model available for calculating the emission of VOCs from each unit operation facility [64,65]. The properties of the wastewater treatment units such as covered and uncovered sump, greatly affected the VOC emission rate. Wastewater capacity, temperature, total dissolved solids (TDS), total suspended solids (TSS), and aqueous VOC components and concentrations were also parameters that were determined. Moreover, to mitigate VOC emission, wastewater treatment facilities should be modified to the closed system or a sealed container. VOC losses from storage tanks can occur from both the standing losses and withdrawing losses when the liquid level changes in the tank [66]. Therefore, understanding of the major contributors and source characteristics are most important for proper management of the emissions in conjunction with considering installation of highly efficient recovery or treatment devices for those major emission sources.

### 4.2. Emission Factors

Based on the emission inventory described above, we developed the specific emission factors of total VOCs for individual sources and the entire petrochemical industry. Results were expressed in units of gram of total VOCs per kg of product (kg VOCs/kg product). Results were compared with other studies associated with the production of VOCs and are presented in Table 3. It was found that the emission factor developed in this study was 0.07 g VOCs/kg product. Among all sectors, combustion stack had the highest value of emission factor of 0.033 g VOCs/kg product, accounting for 51% of total emissions. As for storage tanks and wastewater treatment units, the emission factors were 0.016 and 0.015 g VOCs/kg product, accounting for 25% and 24% of total emissions, respectively. Combustion had the highest emission factor, which could be due to the presence of the large content of organic compounds which were burned. Therefore, reducing the organic content of waste gas before it is sent to the combustion source should be considered to minimize these emissions. This task can be accomplished by installation of recovery or control devices.

The emission factors obtained by this study were lower than those of other studies as shown in Table 4. They were about 8 times lower than the emission factors from crude oil and natural gas extraction and about 28 and 8 times lower than emissions from petroleum refining and chemical raw material manufacturing, respectively. Compared to coating and ink production, the emissions from the aromatic manufacturing were considerably lower than those from paint production and primary plastic manufacturing (more than 100 times). This is probably due to the intensive utilization and modernization of the pollution control devices of the aromatic plant. Furthermore, the use of carcinogenic substances as raw materials as well as manufacturing products led to more stringent control of VOCs as compared with other general VOCs. [67,68].

### 4.3. Spatial Dispersion

#### 4.3.1. Concentrations at the Receptor Sites

**Figure 4:**
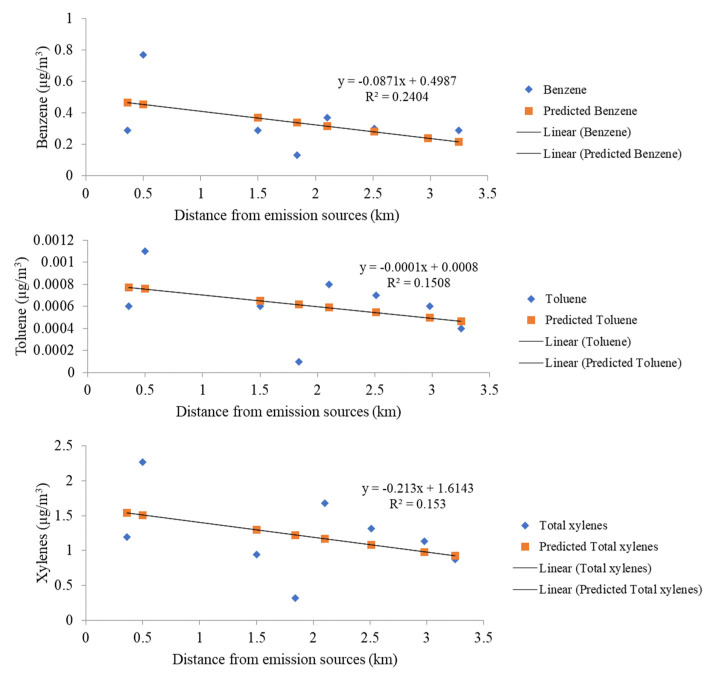
Linear regression analysis between the BTX concentration and the distance from sources.

The spatial dispersion of VOC concentration was modeled using the AERMOD dispersion model and was illustrated as a pollution map of VOC concentration for individual species. Predicted annual average concentrations at the receptors were as presented in Table 5. These modeled concentrations ranged from 0.13 to 0.77 µg/m^3^. Predicted values were within the Thai standard for benzene in the ambient air at 1.7 µg/m^3^ (annual). The predicted average annual concentration of xylenes and toluene ranged from 0.32 to 2.27 and 0.0001 to 0.0011 µg/m^3^, respectively, as shown in Table 6. Compared to other receptor sites, BG was the place where the highest BTX annual concentration was observed, accounting for 0.77, 0.0011, and 2.27 µg/m^3^, respectively. This was probably because its location is closed to the industrial complex. It should be noted that even though NP was the closest receptor site to the industrial source, the BTX concentration at NP quite low because the location of NP was not facing the prevailing wind direction. For receptor sites located downwind of the prevailing wind (south and southwest of study domain), the BTX concentrations at BB, HP, MY, and BP have quite similar values; benzene ranged from 0.24 to 0.37 µg/m^3^, toluene was 0.0006–0.0008 µg/m^3^, and xylenes ranged from 0.94 to 1.68 µg/m^3^. The simple and multiple linear regression models as well as the correlation method are extensively used to reflect the direct effect of air dispersion factors [72,73]. The relationship between the measured ambient concentration and the distance from the source was further evaluated through a correlation analysis. A significant negative correlation was found between the ambient VOC concentration and average distance from emission sources (km) (r = 0.490, *p* < 0.01; 1 d.f. of benzene, r = 0.388, *p* < 0.01; 1 d.f. of toluene, and r = 0.391, *p* < 0.01; 1 d.f. of xylene). These generally implied that the longer the distance from the emission source, the lower the ambient VOC concentration (Figure 4). However, other factors influenced the measured concentrations at each receptor, such as the location being either up- or downwind from the industrial complex and the influence from the local (on-site) activities potentially emitting VOCs at each sampling sites.

#### 4.3.2. Concentration at the Maximum Concentration

**Figure 5:**
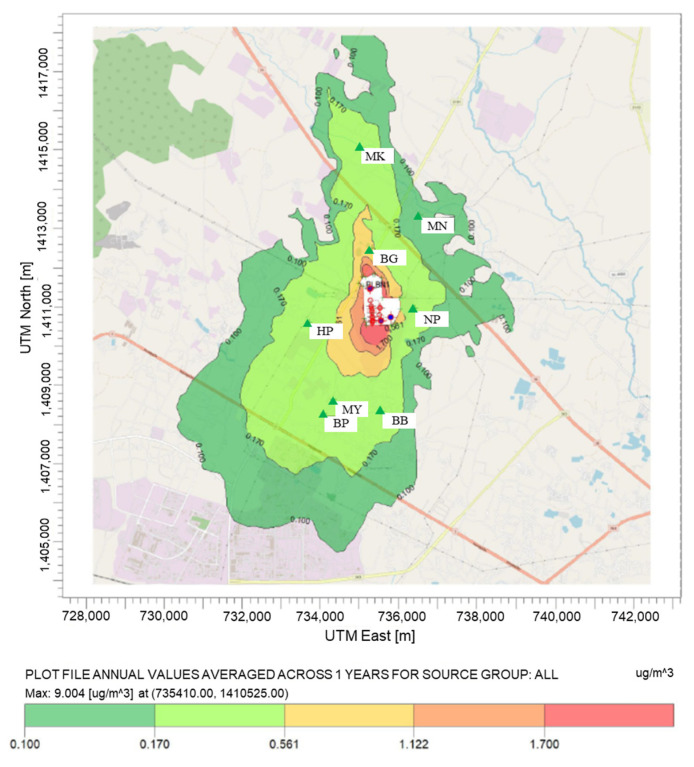
Spatial distribution of average annual benzene concentration.

**Figure 6:**
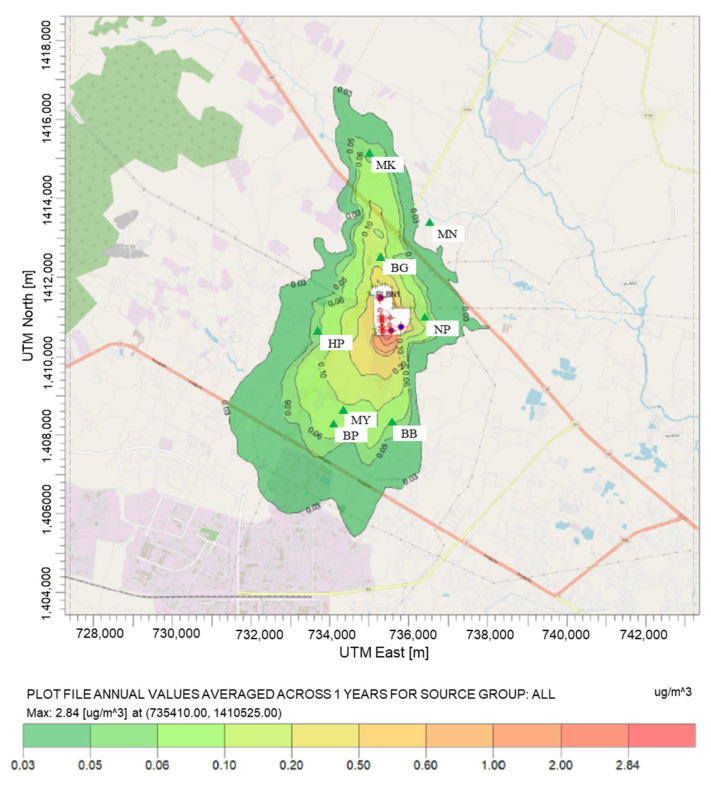
Spatial distribution of average annual toluene concentration.

**Figure 7:**
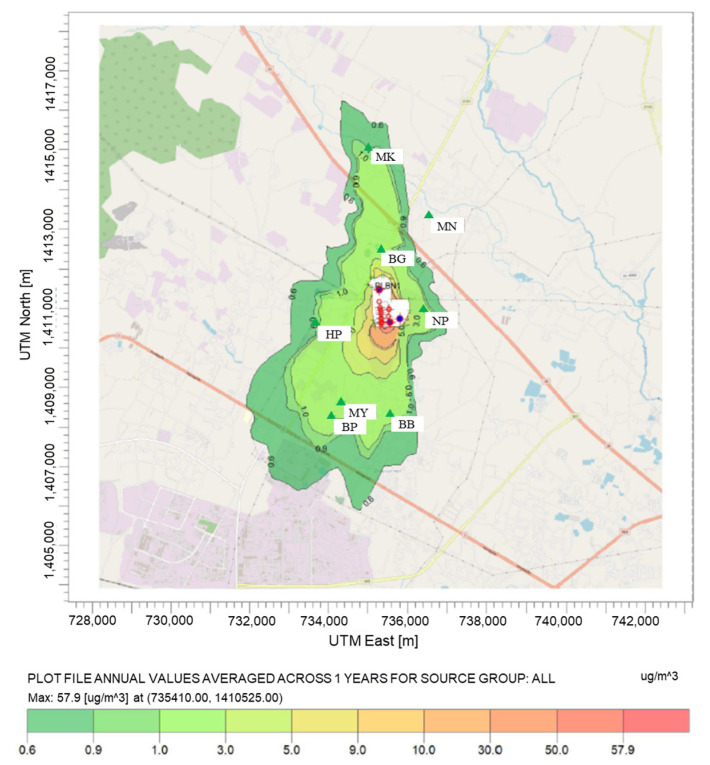
Spatial distribution of average annual xylene concentration.

From the pollution map of benzene, toluene, and xylenes depicted in Figure 5, Figure 6 and Figure 7, the highest average annual concentration of all species was found close to the plant boundary (at UTM: 735410.00 m N, 1410525.00 m E) at the level of 9.00, 2.84, and 57.93 µg/m^3^ for benzene, toluene, and xylenes, respectively. Xylenes were revealed to be the most abundant species in industrial concentration at receptor and maximum ground-level locations, followed by benzene and toluene.

As shown in the figure, the contour maps illustrated that the industrial concentrations were in the downwind direction and dispersed from ground-level sources with low exit velocity. Dispersion characteristics were influenced by thermal buoyancy (buoyancy flux) more than mechanical momentum (momentum flux) [74]. From that information, it can be inferred that the sources with the largest effect on the VOC concentration are ground-level sources. These suspected sources were fugitive diffusion processes such as wastewater treatment units, storage tanks, and fugitive sources. According to the previous study in Thailand, estimating the concentrations of air pollutant from the lower 1.5 sources by using the AERMOD model caused high concentrations near the pollution sources [22,75].

### 4.4. Percentage of Contribution of BTX at Different Receptor Sites

**Figure 8:**
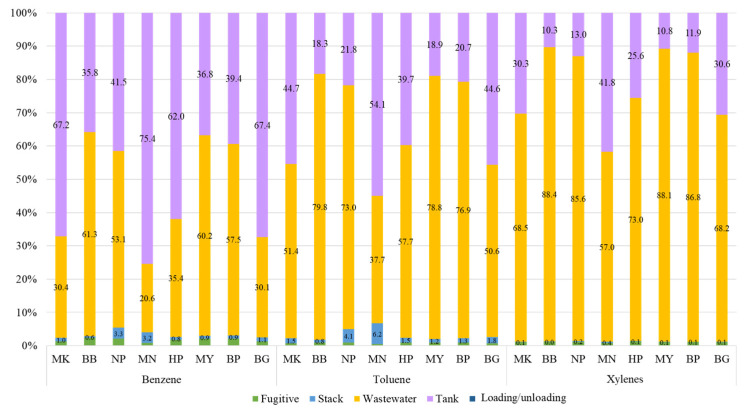
Weighted percentages (%) of source contribution to predicted concentration.

The weighted percentages of contribution from each source group, including combustion stack, storage tanks, loading/unloading, fugitive sources, and WWTs, to the predicted concentration at each receptor are presented in Figure 8. Results from the source contribution analysis revealed that wastewater treatment units were the dominant contributor, with a contribution of 20.6–88.4%, followed by storage tanks (10.3–75.4%) for BTX concentration. On the other hand, other emission sources such as combustion stacks, fugitive sources, and loading/unloading activities were not the important sources contributing to the ground level concentrations of BTX in this study.

An intensive study was carried out for wastewater treatment units to elaborate the major unit contributing to the VOC concentration. The results from the WATER9 model (Supplementary Materials, Table S7) demonstrated that benzene, toluene, and xylene emissions were mostly contributed to by the effluent open sump unit (XC-23), accounting for 37.98%, 46.48%, and 55.59% of the all-wastewater treatment units, respectively. These emissions could be reduced by enclosing the sump in a closed system. Based on previous research, covering the basin dramatically decreased VOC concentrations [62,76,77]. On the other hand, storage tanks also comprised a large proportion of the contributors to BTX concentration; therefore, reducing the ambient concentration should be considered. From the previous studies, implementing vapor recovery units and improving tank conditions were the methods of interest to reduce emissions and concentrations from storage tanks [78,79].

### 4.5. Mitigation Scenario Analysis

**Figure 9:**
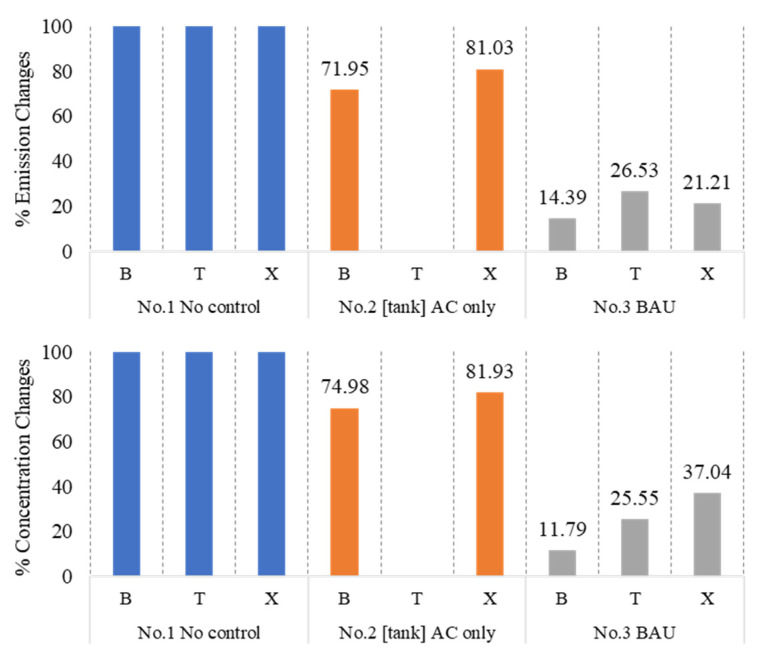
Success of implemented technology in past and current situations.

Figure 9 depicts the success of implemented measures aimed at reducing emissions and ambient concentration through scenarios No. 1–3. As for the current scenario (BAU), after installation of the vapor recovery unit (VRU), emission of BTX was reduced by 73.47–85.61% and was effective in reducing the average concentration of these chemicals by 62.96–88.22% from their original values.

**Figure 10:**
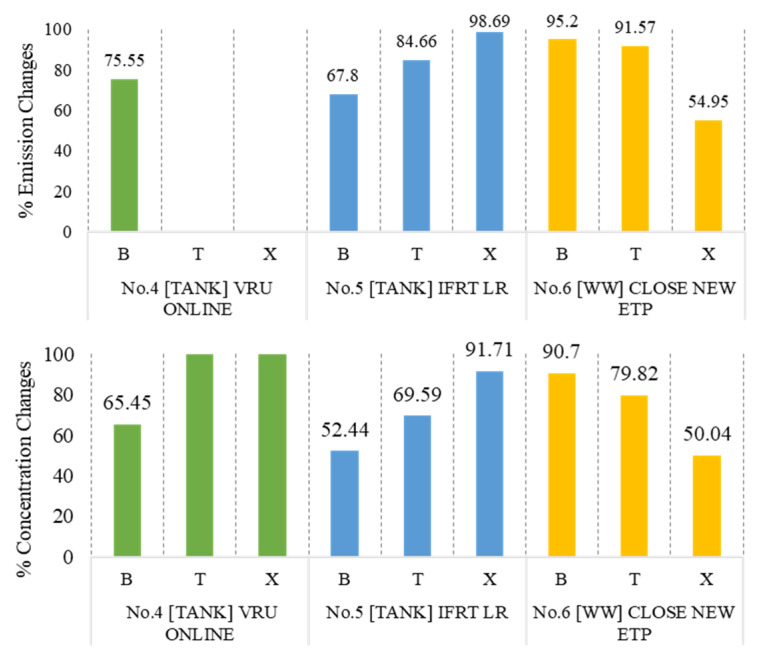
Mitigation measure analysis.

Quantitative analysis of mitigation measures is illustrated in Figure 10. The percentage of emissions and concentration as compared to the business-as-usual (BAU/scenario No. 3) case in different scenarios is presented. The patterns of emission and concentration reduction were found to be inconsistent among each mitigation measure due to the relationship between the target sources of measurement and substances. For example, scenario No. 5 can reduce industrial emissions to 1.31–32.20%, while the average ground-level concentration can be reduced to 1.29–47.56%. In this scenario, benzene emissions and concentrations were significantly reduced compared to toluene and xylenes. These results are consistent with other research, in which improving the tank characteristics and conditions resulted in the decrease in VOC emissions and concentration by 49% and 26%, respectively [78]. On the contrary, xylenes and toluene were notably reduced in emissions and concentrations in scenario No. 6. In this scenario, the industrial emissions were reduced by 4.80–45.05%, and the ground level concentrations could be reduced by 9.30–49.96%. These findings are similar to the previous study, in which covering the open area of the wastewater treatment unit reduced the emission and ambient concentrations by more than 27% [65]. However, implementing scenario No. 4 only affects benzene emissions and concentrations, reducing them to 24.45% and 34.97%, since this scenario is only related to benzene. Therefore, to reduce the emissions and concentrations of benzene in the environment, scenario No. 5 should be given priority. On the other hand, reduction in the emissions and concentrations of xylenes will be greatly affected when scenario No. 6 is implemented.

## 5. Conclusions

In this study, a BTX emission inventory and emission factors from six different sources in an aromatics petrochemical industry were comprehensively developed. Results from the emission inventory revealed that xylenes were the dominant species, accounting for 62% of BTX emissions, followed by benzene (30%) and toluene (8%). Dominant emission sources of toluene and xylenes were wastewater treatment units and storage tanks, while combustion stacks were the largest contributors of benzene emissions. The AERMOD dispersion model was applied to support the source contribution to the ambient concentration analysis. Modeled results showed that the maximum ground level concentration of BTX was found at the plant boundary due to the diffusion mechanism of the major source. However, the concentrations at the interesting receptors do not exceed the Thai standard for benzene. Results from the source contribution to concentration at receptors indicated that wastewater treatment and storage tanks were the major sources affecting the ground level concentration. Consequently, the scenario analysis was set to evaluate the magnitude of success of emissions and concentration control strategies. Results showed that emission and ambient concentrations were reduced by 88%, 75%, and 63% of benzene, toluene, and xylenes. The prediction of mitigation measures indicated that the improvement of tank characteristics and covering the wastewater containers were potential measures to reduce the emissions and concentration of BTX. In order to control and reduce VOC emissions and concentration, moreover, the proper management of production processes, such as the air/fuel ratio and the catalyst, should be taken into consideration.

The main contribution of this work is the study of source contribution and spatial distribution of VOCs from a petrochemical industry. The methodology demonstrated in this study can be applied and used for comparison purposes with other studies as well as implementation in other polluted areas, particularly in the petroleum and petrochemical industries. The methodology and data interpretation demonstrated in this study can be used as a guideline for investigating emission and dispersion of air pollutants from the industrial sectors and can lead to the determination of appropriate measures for various industrial sectors in different areas. The emission factors obtained from this study are also useful for the development of an emission inventory of the petrochemical industry.

However, this study characterized the BTX emissions and concentrations from the petrochemical industry in the petroleum and petrochemical industrial complex with direct in situ measurements and predicted their propagations using an air quality model. It is analyzed based on the assumption of a constant emission rate, which is the limitation of this study.

## 6. Recommendations

For further study, aside from implementing the best available technology and constructing the control equipment, proper management of the fuel-to-oxygen ratio in combustion units and installing a catalyst may be a possible solution to reduce the VOC concentration and emission from the combustion processes [63].

## Figures and Tables

**Table 1:** Measured BTX concentrations (ppbv) in the different regions.

Regions	BTX Concentrations (ppbv)			References
	Benzene	Toluene	Xylenes	
Industrial region				
Rayong, Thailand	6.35	0.28	10.98	[19]
Lanzhou, China	4.48	4.00	4.40	[27]
Taiwan	1.85	2.20	8.10	[28]
Shiohama, Japan	0.40	1.60	1.30	[29]
Aliaga, Turkey	1.47	1.03	0.63	[30]
Urban region				
Bangkok, Thailand	0.35	2.47	0.41	[31]
Hong Kong	1.64	4.45	1.26	[32]
London, UK	0.80	2.01	1.34	[32]
Lanzhou, China	1.94	1.01	2.08	[27]
Rural region				
Aliaga, Turkey	0.71	1.23	0.27	[30]

**Table 2:** Emission estimating techniques for each emission source.

Source Category	Data Collection	Estimation Technique
Combustion stack	Measured concentration of VOCs from stack	Direct measurement
Fugitive	Measured concentration of VOCs from each part	Correlation equation
Storage tank	Consumption and operation reports	Model/formula
WWT	Measured concentration of VOCs from the wastewater treatment unit	Model/formula
Loading/unloading	Consumption and operation reports	Direct measurement

**Table 3:** Detailed scenario analysis.

Scenarios		Scenario Details
Past		
No. 1	No control	No control technologies
No. 2	(TANK) AC only	Activated carbon installation
Current situation		
No. 3	BAU	Business as usual; vapor recovery unit (VRU) installation
Mitigation measures		
No. 4	(TANK) VRU ONLINE	Directly measure on monitoring station at the VRU
No. 5	(TANK) IFRT LR	Improving the tank characteristics
No. 6	(WW) CLOSE NEW ETP	Enclosing the open sump

**Table 4:** Comparison of emission factors with other studies.

Source	Activity Data	Emission Factor	Unit	Reference
Crude oil and natural gas extraction	Crude oil exploration	1.5275	g VOCs/kg product	[69]
	Natural gas exploration	0.5	g VOCs/kg product	[69]
Petroleum refining	Crude oil processing volume	1.82	g VOCs/kg product	[69]
	Methanol production	5.55	g VOCs/kg product	[70]
Chemical raw material manufacturing	Benzene production	0.55	g VOCs/kg product	[70]
	Ammonia synthesis	4.72	g VOCs/kg product	[69]
Coating production	Paint production	81.4	g VOCs/kg product	[69]
Ink production	Production of primary form plastic	50	g VOCs/kg product	[71]
This study	Aromatics production	0.07	g VOCs/kg product	
	Combustion stacks	0.033		
	Storage tanks	0.00030		
	Loading/unloading	0.016		
	Fugitive sources	0.0000031		
	WWTs	0.015		

**Table 5:** Information for the receptors in the modeling domain.

Receptor	Average Distance from Emission Sources (km)	Direction from Emission Sources
MK	3.25	N
BB	2.98	S
NP	0.36	SE
MN	1.84	NE
HP	1.50	SW
MY	2.10	S
BP	2.51	SW
BG	0.50	N

**Table 6:** Predicted concentrations at the receptors.

Receptors	Annual Concentration (µg/m^3^)		
	Benzene	Toluene	Total Xylenes
Maximum ground level concentration	9.00	2.84	57.93
MK	0.29	0.0004	0.87
BB	0.24	0.0006	1.13
NP	0.29	0.0006	1.19
MN	0.13	0.0001	0.32
HP	0.29	0.0006	0.94
MY	0.37	0.0008	1.68
BP	0.30	0.0007	1.31
BG	0.77	0.0011	2.27

## Data Availability

Not applicable.

## Supplementary Materials

The following supporting information can be downloaded at: https://www.mdpi.com/article/10.3390/ijerph20032280/s1, Table S1: Air dispersion models; Table S2: Equipment leak rate and screening value of synthetic organic chemical manufacturing industry (SOCMI); Table S3: Saturation (S) factors for calculating petroleum liquid loading losses; Table S4: The predicted annual concentration of benzene; Table S5: The predicted annual concentration of toluene; Table S6: The percentage of emission reduction and annual concentration and its percentage of concentrations reduction of xylenes; Table S7: VOCs emissions from each unit of wastewater treatment unit. References [80,81,82,83,84,85,86,87,88] are cited in the supplementary materials.
