# Supplementary material for: Integrated Sustainable Management of Petrochemical Industrial Air Pollution
Source: Int J Environ Res Public Health. 2023 Jan 27;20(3):2280. doi: 10.3390/ijerph20032280 (PMC9914942; doi:10.3390/ijerph20032280)
Supplement: Supplementary file 1 [file ijerph-20-02280-s001.zip › ijerph-2155107-supplementary.pdf]

Supplementary Materials:

Integrated sustainable management of petrochemical industrial air pollution

Jutarat Keawboonchu [1,2], Sarawut Thepanondh [1,2,*], Vanitchaya Kultan [1,2], Nattaporn Pinthong [1,2], Wissawa Ma-lakan [1,2] and Mark Gregory Robson [3]

1 Department of Sanitary Engineering, Faculty of Public Health, Mahidol University, Bangkok 10400 Thailand; jutarat.kboonchu@gmail.com (J.K.); sarawut.the@mahidol.ac.th (S.T.); vanitchayakultan@gmail.com (V.K.), nattasan62@gmail.com (N.P.), wissawamalakan@gmail.com (W.M.)
2 Center of Excellence on Environmental Health and Toxicology (EHT), OPS, MHESI, Thailand
3 School of Environmental and Biological Science, Department of Plant Biology, Rutgers, The State University of New Jersey, New Brunswick, New Jersey, USA
* Correspondence: sarawut.the@mahidol.ac.th; Tel.; 0 2354 8540

Table S1. Air dispersion models.

Modelling types	Model descriptions	Advantage	Limitation
AERMOD [1-2]	AERMOD model, a steady state plume model based on planetary boundary layer turbulence structure and scaling scheme, can be applied to surface and elevated sources as well as simple and complex topography with a prediction range of less than 50 km.	The model is widely used in various applications, including pollutant concentrations and applications for odor nuisance. The model can generate the concentration contributions of different sources of air pollutants.	The model requires external validation and resolves background concentrations.
CALPUFF [3-4]	CALPUFF model, an advance non steady state puff dispersion model, can process for multi-layer and multi-species of air pollutants to simulate the influences of spatiotemporal changes on the migration, transformation, and	The model can be used to simulate a medium- to large-scale rough and complex topographical domain, and its meteorological models include onshore and aquatic	The completely hourly meteorological data is considerably required to process the model.

Modelling types	Model descriptions	Advantage	Limitation
	removal of pollutants with the recommended distance range above 50km.	boundary layer modules.	
HYSPLIT [5-6]	HYSPLIT model is a computing air parcel trajectory and dispersion model using either puff or particle approaches. The model tracks the movement if partical and gases carried by air flow.	The model can be used to compute and predict the regional influence for atmospheric vertical profile using forward and backward trajectory.	The application of multi-emission sources and the complicated emission rates are still limitation.
CAMx [7]	CAMx is the photochemical grid model used to simulate physical and chemical processes governing the formation and transport of ozone and particulate matter on multi-scales in cities and regions.	The model can simulate air quality on multiple geographic scales. The model can also be used for sensitivity, source contribution and process analysis.	The uncertainty of emission inventories is susceptible to the source apportionment analysis. The application of The CAMx surface model still has some limitations.
CMAQ [8-9]	CMAQ model, a multi-pollutant air quality modeling system, can simulate particulate matter, ozone, visibility, toxic airborne pollutants, and nutrient pollutant species overall the troposphere.	The model can process in scenario of multi-dimensional information at the same time and makes the air quality simulation technology to be efficient and accurate.	The sophisticated computer software and hardware equipment is required. Many specific input data is required such as weather data from MM5 and WRF model.

Table S2. Equipment leak rate and screening value of synthetic organic chemical manufacturing industry (SOCMI)[a].

Equipment type	Default zero emission rate (kg/hr./item)	Pegged emission rates (kg/hr./item)	Correlations[b] (kg/hr./item)
Gas/vapor valve	0.00000066	0.11	$= 1.87 \times 10^{-6} x(SV)^{0.873}$
Light liquid valve	0.00000049	0.15	$= 6.41 \times 10^{-6} \times (SV)^{0.797}$
Pump	0.0000075	0.62	$= 1.90 \times 10\text{-}5 \times (SV)^{0.824}$
Compressor	0.0000075	0.62	$= 1.90 \times 10\text{-}5 \times (SV)^{0.824}$
Pressure relief valve	0.0000075	0.62	$= 1.90 \times 10^{-5} \times (SV)^{0.824}$
Agitators	0.0000075	0.62	$= 1.90 \times 10\text{-}5 \times (SV)^{0.824}$
Connector/flange	0.00000061	0.22	$= 3.05 \times 10\text{-}6 \times (SV)^{0.885}$

Notes: kg/hr./source = kilograms TOC per hour per source.
a: Data reported in USEPA (1995). For closed sampling points, if the sampling bottle is connected to the sampling port, use the emission factor of the "connector"; if the sampling bottle is not connected to the sampling port, use the emission factor of the "open-ended line".
b: SV is the net screening value (SV, ppmv) measured by the monitoring device.
c: The light liquid pump factors can also be applied to compressors, pressure relief valves, agitators, and heavy liquid pumps.

Table S3. Saturation (S) factors for calculating petroleum liquid loading losses.

Cargo carrier	Mode of operation	S factor
**Tank tracks and**	Submerged loading of a clean cargo tank	0.50
**rail tank cars**	Submerged loading: dedicated normal service	0.60
	Submerged loading: dedicated vapor balance service	1.00
	Splash loading of a clean cargo tank	1.45
	Splash loading: dedicated normal service	1.45
	Splash loading: dedicated vapor balance service	1.00
**Marine vessels**	Submerged loading: ships	0.2
	Submerged loading: barges	0.5

Table S4. The predicted annual concentration of benzene.

Scenario No.	Receptor sites	Annual Concentration (μg/m³)
**Scenario No. 1**	MK	3.509
	BB	1.482
	NP	2.527
	MN	2.051
	HP	2.334
	MY	2.137
	BP	1.852
	BG	12.745
**Scenario No. 2**	MK	2.494
	BB	1.184
	NP	2.021
	MN	1.532
	HP	1.734
	MY	1.663
	BP	1.424
	BG	8.298
**Scenario No. 3**	MK	0.294
	BB	0.244
	NP	0.287
	MN	0.132
	HP	0.291
	MY	0.368
	BP	0.296
	BG	0.770
**Scenario No. 4**	MK	0.174
	BB	0.187
	NP	0.204
	MN	0.064
	HP	0.173
	MY	0.278
	BP	0.218
	BG	0.459

Scenario No.	Receptor sites	Annual Concentration (µg/m³)
**Scenario No. 5**	MK	0.117
	BB	0.166
	NP	0.183
	MN	0.043
	HP	0.129
	MY	0.247
	BP	0.191
	BG	0.303
**Scenario No. 6**	MK	0.275
	BB	0.212
	NP	0.255
	MN	0.126
	HP	0.269
	MY	0.321
	BP	0.259
	BG	0.720

Table S5. The predicted annual concentration of toluene.

Scenario No.	Receptor sites	Annual Concentration (μg/m^3)
**Scenario No. 1**	MK	0.412
	BB	0.148
	NP	0.257
	MN	0.182
	HP	0.218
	MY	0.254
	BP	0.220
	BG	1.056
**Scenario No. 2**	MK	-
	BB	-
	NP	-
	MN	-
	HP	-
	MY	-
	BP	-
	BG	-
**Scenario No. 3**	MK	0.057
	BB	0.061
	NP	0.068
	MN	0.023
	HP	0.058
	MY	0.092
	BP	0.072
	BG	0.150
**Scenario No. 4**	MK	-
	BB	-
	NP	-
	MN	-
	HP	-
	MY	-
	BP	-
	BG	-

Scenario No.	Receptor sites	Annual Concentration (μg/m³)
**Scenario No. 5**	MK	0.033
	BB	0.051
	NP	0.055
	MN	0.012
	HP	0.037
	MY	0.076
	BP	0.058
	BG	0.088
**Scenario No. 6**	MK	0.048
	BB	0.046
	NP	0.052
	MN	0.021
	HP	0.047
	MY	0.069
	BP	0.055
	BG	0.126

Table S6. The percentage of emission reduction and annual concentration and its percentage of concentrations reduction of xylenes.

Scenario No.	Receptor sites	Annual Concentration (μg/m³)
**Scenario No. 1**	MK	4.729
	BB	2.054
	NP	2.274
	MN	1.673
	HP	1.942
	MY	3.528
	BP	3.094
	BG	17.759
**Scenario No. 2**	MK	3.787
	BB	1.728
	NP	1.872
	MN	1.326
	HP	1.663
	MY	2.928
	BP	2.538
	BG	14.026
**Scenario No. 3**	MK	0.873
	BB	1.131
	NP	1.191
	MN	0.317
	HP	0.943
	MY	1.684
	BP	1.310
	BG	2.274
**Scenario No. 4**	MK	-
	BB	-
	NP	-
	MN	-
	HP	-
	MY	-
	BP	-

Scenario No.	Receptor sites	Annual Concentration (µg/m³)
	BG	-
**Scenario No. 5**	MK	0.858
	BB	1.125
	NP	1.182
	MN	0.309
	HP	0.928
	MY	1.673
	BP	1.300
	BG	2.233
**Scenario No. 6**	MK	0.485
	BB	0.482
	NP	0.529
	MN	0.200
	HP	0.496
	MY	0.721
	BP	0.571
	BG	1.268

Table S7. VOCs emissions from each unit of wastewater treatment unit.

Unit code	Air emission rate (g/s)		
	Benzene	Toluene	Xylenes
waste drop from pipe to bio eq	0.0239	0.00814	0.00488
waste drop from pipe to storm DAF	3.2E-12	6.8E-12	7.23E-10
XC12 Bio tranfer	0.00236	0.000635	0.0132
ME05 DAF	1.35E-12	3.28E-13	4.77E-10
aeration tank ME12A	0.0112	0.00248	0.0326
aeration tank ME12B	0.0114	0.00241	0.0312
Clearifier ME15A	1.17E-05	2.25E-06	5.97E-05
Clearifier ME15B	0.000012	2.32E-06	6.27E-05
ME101A DAF	9.24E-05	2.17E-05	0.000218
XC20 final basin	2.01E-05	3.9E-06	0.000172
ME101B DAF	9.39E-05	2.23E-05	0.000227
XC19 open sump	2.29E-05	7.17E-06	0.000279
New ETP_TK24	3.66E-18	1.46E-18	6.78E-17
New ETP_contact tank (XC23)	0.0306	0.0122	0.3
New ETP_Flocculation tank (XC26)	0.000855	0.000323	0.155
New ETP_Settling zone (XC27)	3.3E-07	1.01E-07	0.00175

References

1. EPA, U. User's Guide for the AMS/EPA Regulatory Model (AERMOD). 2013. Available online: https://gaftp.epa.gov/Air/aqmg/SCRAM/models/preferred/aermod/aermod_userguide.pdf (accessed on 24 December 2022).
2. Kumar, A.; Patil, R. S.; Dikshit, A. K.; Kumar, R. Application of AERMOD for short-term air quality prediction with forecasted meteorology using WRF model. *Clean Technol. Environ. Policy* **2017**, 19 *(7)*, 1955-1965. DOI: 10.1007/s10098-017-1379-0
3. EPA, U. Documentation of the Evaluation of CALPUFF and Other Long Range Transport Models Using Tracer Field Experiment Data. Available online: https://www.epa.gov/sites/default/files/2020-10/documents/epa-454_r-12-003_0.pdf (accessed on 24 December 2022).
4. Joseph, S. S., David G. S., Robert J. Y. A User’s Guide for the CALPUFF Dispersion Model (Version 5). 2000. Available online: https://www.eoas.ubc.ca/courses/atsc507/ADM/calpuff/CALPUFF_UsersGuide-v5-excellent.pdf (accessed on 25 December 2022).
5. Roland D., Barbara S., Glenn R., Ariel S., Albion T., Sonny Z., Chris L., Alice C. HYSPLIT USER's GUIDE. 2022. Available online: https://www.arl.noaa.gov/documents/reports/hysplit_user_guide.pdf (accessed on 25 December 2022).
6. NOAA. HYSPLIT. Available online: https://www.arl.noaa.gov/hysplit/ (accessed on 25 December 2022).
7. Ramboll E. H. User’s Guide Comprehensive Air Quality Model With Extensions Version 7.10. 2020. Available online: https://camx-wp.azurewebsites.net/Files/CAMxUsersGuide_v7.10.pdf (accessed on 25 December 2022).
8. Appel, K. W.; Napelenok, S. L.; Foley, K. M.; Pye, H. O. T.; Hogrefe, C.; Luecken, D. J.; Bash, J. O.; Roselle, S. J.; Pleim, J. E.; Foroutan, H.; Hutzell, W. T.; Pouliot, G. A.; Sarwar, G.; Fahey, K. M.; Gantt, B.; Gilliam, R. C.; Heath, N. K.; Kang, D.; Mathur, R.; Schwede, D. B.; Spero, T. L.; Wong, D. C.; Young, J. O. Description and evaluation of the Community Multiscale Air Quality (CMAQ) modeling system version 5.1. *Geosci. Model Dev.* **2017**, 10 *(4)*, 1703-1732. DOI: 10.5194/gmd-10-1703-2017.
9. EPA, U. Community Multiscale Air Quality Modeling System (CMAQ). Available online: https://www.epa.gov/cmaq/cmaq-models-0 (accessed on 25 December 2022).
